# POLICIES AND PREVENTION OF U.S. WOMEN’S VIOLENT DEATH ACROSS AGES

**DOI:** 10.1093/geroni/igz038.1782

**Published:** 2019-11-08

**Authors:** Sonia L Salari, Carrie Sillito

**Affiliations:** University of Utah, Salt Lake City, Utah, United States

## Abstract

U.S. violent death rates (homicide and suicide) are the highest in the developed world. Of all female murders (femicide), the majority are male perpetrated, intimate partner violence (IPV- 55-63%). Men are more often killed and by other male acquaintances, with only 2.8% IPV. Proportionally, older women (50+) have the top homicide victim rate (26%) among women. The baby boom cohort has been suicidal and aging has exacerbated the problem. Women are less likely to kill themselves, and the methods differ. We ask are mid and later life women’s lethal victimization similar to younger women? What are policy implications for prevention? Our research uses national level data from news surveillance of 728 intimate partner homicide suicide (IPHS) events and the State Firearm Law Database (SFLD) to improve our understanding of violent cause mortality by sex, age, method and location. IPHS patterns show 90% of events used firearm and 90% were male perpetrated. Results of multivariate analyses show young women had greater awareness and fear before IPHS. Evidence finds older men sometimes decided to kill their IP as part of their own suicide, without a history of known domestic violence. Older women have disproportionately low use of shelters, police and protective orders. SFLD shows population adjusted states with more DV firearms laws have significantly fewer IPHS events. Firearm culture has restricted research, blocked law enforcement and has done little to reduce gun access in households with vulnerable populations (e.g., suicidal husbands). Lethality Assessment Protocols could be modified for elder women’s unique situation.

